# Supplementary material for: Comparative investigation of bagging enhanced machine learning for early detection of HCV infections using class imbalance technique with feature selection
Source: PLoS One. 2025 Jun 26;20(6):e0326488. doi: 10.1371/journal.pone.0326488 (PMC12201643; doi:10.1371/journal.pone.0326488)
Supplement: Attachment — Submitted filename: Response_to_Reviewers_Plos_one.docx [file pone.0326488.s001.docx]

Article Title: " Comparative Investigation of Bagging Enhanced Machine Learning for Early Detection of HCV Infections Using Class Imbalance Technique with Feature Selection"

Paper ID: PONE-D-25-00479

To

Editor,

PLOS ONE

Subject: Response to reviewers

Dear Editor,

We would like to express our gratitude for giving us the opportunity to submit a revised draft of our manuscript titled " Comparative Investigation of Bagging Enhanced Machine Learning for Early Detection of HCV Infections Using Class Imbalance Technique with Feature Selection". We appreciate the time and effort that you and the reviewers have dedicated to providing valuable feedback on our manuscript. We are grateful to the reviewer for his/her insightful comments on our manuscript. We have been able to incorporate changes to reflect most of the suggestions provided by the reviewer.

We are uploading (a) our point-by-point response to the comments (below) (response to reviewers) which we update in the final manuscript.

Best regards,

Assoc. Prof. Dr. Mohd Arfian Ismail

Here is a point-by-point response to the associate editor and reviewers’ comments and concerns.

**Associate Editor Comment:**

| **Associate Editor Comment-1** | The implementation is lacking in clarity. The authors must rework this section and give more clarity on the materials and methods. In addition, address the reviewers' completely. |
| --- | --- |
| **Author Response-1** | Thank you for your valuable feedback. We have carefully revised the implementation section to enhance clarity and provide a more detailed explanation of the materials and methods. Additionally, we have thoroughly addressed all reviewer comments to ensure a comprehensive and well-structured presentation. We appreciate your insights and believe these improvements will strengthen the manuscript. Page: 6,7 Line: 233-288 |

**Reviewer’s Comment (Reviewer #1):**

| **Reviewer’s Comment-1** | The abstract is well written. However, the introduction seems to suffer from the lack of one topic, and that is a brief definition of artificial intelligence and machine learning and its applications in the field of healthcare. For this section, you can refer to more studies and applications of machine learning in various fields of health. You can also use the following studies. "Artificial intelligence in drug discovery and development against antimicrobial resistance: A narrative review" "Mobile apps for COVID-19 detection and diagnosis for future pandemic control: Multidimensional systematic review" Using these studies can provide important resources of machine learning in the field of healthcare. |
| --- | --- |
| **Author Response-1** | Thank you for your insightful feedback. We appreciate your suggestion and have incorporated the recommended references to strengthen the discussion on artificial intelligence and machine learning applications in healthcare. These additions enhance the related work section, providing a broader perspective on the topic. Page: 4 Line: 112 |
| **Reviewer’s Comment-2** | Regarding the choice of these algorithms, you need to explain why you used the bugging algorithms at all? The rest of the issues seem to be well written and presented clearly and do not need any special changes and can be accepted as is. |
| **Author Response-2** | Thank you for your insightful feedback. We have now included a detailed explanation of our choice of bagging algorithms. Bagging (Bootstrap Aggregating) is employed in our study due to its ability to enhance model stability, reduce variance, and mitigate the risk of overfitting, particularly in complex datasets. By aggregating multiple base models trained on different subsets of data, bagging improves generalization and robustness. We have added this explanation to clarify our methodology and strengthen the justification for our approach. Page: 3 Line: 64-83 |

**Reviewer’s Comment (Reviewer #2):**

| **Reviewer’s Comment-1** | The paper lacks a clear explanation of why certain features were selected or removed based on the ANOVA F-test results, especially regarding the clinical relevance |
| --- | --- |
| **Author Response-1** | Thank you for your valuable feedback. In the revised version, we have provided a clearer explanation of the feature selection process using the ANOVA F-test, emphasizing the clinical relevance of the selected features for HCV classification. This addition enhances the transparency of our methodology and ensures a stronger justification for the chosen features. Page: 23 Line: 605-627 |
| **Reviewer’s Comment-2** | The methodology section lacks detail about the specific parameters used for each machine learning algorithm. |
| **Author Response-2** | Thank you for your feedback. We have addressed this concern by detailing the specific parameters used for each machine learning algorithm in Figures 2-7. Additionally, we have provided a step-by-step explanation of the methodology to clarify how each algorithm operates within our framework. We believe these additions enhance the comprehensiveness and transparency of our methodology section. |
| **Reviewer’s Comment-3** | There is no discussion about the computational complexity or processing time of the different methods. |
| **Author Response-3** | Thank you for your valuable feedback. We have now included a discussion on the computational complexity and processing time of the different methods in Tables 2 and 3. This addition enhances the completeness of our analysis and provides a clearer comparison of the efficiency of the implemented approaches. Page: 39, 42 |
| **Reviewer’s Comment-4** | There is no validation using an external dataset to confirm the robustness of the proposed methods. |
| **Author Response-4** | Thank you for your insightful feedback. While we have evaluated the robustness of the proposed methods using performance metrics, ROC-AUC, and the confusion matrix, we acknowledge the importance of validation with an external dataset. We plan to incorporate this aspect in our future work to further strengthen the generalizability of our approach. |
| **Reviewer’s Comment-5** | The authors do not justify why they chose a 80-20 split for training-testing instead of other common ratios. |
| **Author Response-5** | Thank you for your valuable feedback. We have now provided a justification for selecting the 80-20 training-testing split in the methodology section, explaining its balance between model training and evaluation. This addition enhances the clarity and rationale behind our experimental setup. Page: 7 Line: 270-279 |
| **Reviewer’s Comment-6** | The figures showing confusion matrices (14 and 17) are too small and difficult to interpret. |
| **Author Response-6** | Thank you for your valuable feedback. We have updated Figures 14 and 17 to improve their clarity and readability, ensuring that the confusion matrices are more interpretable. We appreciate your suggestion, as it enhances the overall presentation of our results. Page: 41,44 |
| **Reviewer’s Comment-7** | The paper does not address the potential bias in the dataset regarding demographic factors like age and gender distribution |
| **Author Response-7** | Thank you for your valuable feedback. The discussion on potential bias in the dataset, including demographic factors such as age and gender distribution, was already addressed in our paper. However, based on your comment, we have extended this discussion to provide a more detailed analysis and ensure a comprehensive understanding of its impact on model performance. Page: 24 Line: 645-652 |
| **Reviewer’s Comment-8** | The discussion section does not adequately address the limitations of the study or potential areas for future research. |
| **Author Response-8** | Thank you for your valuable feedback. We have now expanded the discussion section to explicitly address the limitations of the study and outline potential areas for future research. These additions enhance the completeness of our analysis and provide a clearer direction for further exploration. Page: 34,35 Line: 951-957, 985-991 |
| **Reviewer’s Comment-9** | The paper lacks a clear explanation of how the proposed method could be implemented in real-world clinical settings and what resources would be required. |
| **Author Response-9** | Thank you for your valuable feedback. We have now included a discussion on how the proposed method could be implemented in real-world clinical settings, along with the necessary resources required for practical deployment. This addition enhances the applicability of our study and provides a clearer perspective on its real-world impact. Page: 34, Line: 973-985 |
