# Supplementary material for: Comparative investigation of bagging enhanced machine learning for early detection of HCV infections using class imbalance technique with feature selection
Source: PLoS One. 2025 Jun 26;20(6):e0326488. doi: 10.1371/journal.pone.0326488 (PMC12201643; doi:10.1371/journal.pone.0326488)
Supplement: Attachment — Submitted filename: Response_to_Reviewers_Plos_one_auresp_2.docx [file pone.0326488.s002.docx]

Here is a point-by-point response to the associate editor's comments and concerns.

**Associate Editor Comment:**

| **Associate Editor Comment-1** | The authors should recreate the blurred flowcharts, especially figures 2-7. Others can be improved as well. I think it has to do with the tool used. I suggest that you use Visio to create excellent figures. Additionally, minor English editing is required. |
| --- | --- |
| **Author Response-1** | Thank you for your feedback. I have redrawn the flowcharts, including figures 2-7, as well as figures 11 and 12, in response to your suggestion. I believe the updated figures should now meet the clarity standards you were expecting.  Additionally, I made minor revisions to the abstract and conclusion, as requested, to improve the language and readability. |
